# Arsenicum-album

**Published:** 1896-01

**Authors:** 


### HOMŒOPATHIC PHYSICIAN,

A MONTHLY JOURNAL OF

HOMŒOPATHIC MATERIA MEDICA AND CLINICAL MEDICINE.

"If our school ever gives up the strict inductive method of Hahnemann, we are lost, and deserve only to be mentioned as a caricature in the history of medicine."—CONSTANTINE HERING.

Vol. XVI. JANUARY, 1896. No. 1.

### EDITORIAL.

ARSENICUM-ALBUM.—The last editorial in the December number closed with a lot of comparisons upon *starting*, suggested by the pathogenesis of Arsenic. The list, however, was not quite complete as it stands in the editor's note-book. The remaining indications are, therefore, here appended :

Carbo-veg., Kali-hydriodicum, and Silicea have starting at the least noise. Kali-carbonicum, Mercurius, Natrum-muriaticum, Opium, Phosphorus, Silicea, and Tartar-emetic all have great tendency to start.

Borax, starting of limbs by any unusual cry.

Turning now to the other notes given by Dr. Lippe in connection with Arsenicum, we find that Arsenicum has sleeplessness from intense anguish, restlessness, and tossing about; the patient getting out of bed and sitting in a chair for a period of time, then returning to the bed again. This recalls the Aconite condition in which, as stated in previous editorials, the patient is restless during the night from anxiety, fever, and thirst. The Aconite patient rolls about the bed, complains, and drinks much water. Rhus-toxicodendron, the patient is sleepless from pain.

Calcarea-carbonica, sleeplessness from the crowding of thoughts into the mind.

Lachesis, the patient is roused from sleep by the aggravation of his complaint. The Lachesis patient *sleeps into an aggrava-*

*tion.* The Lachesis symptoms are all aggravated by sleep. This is Dr. Guernsey's key-note for Lachesis.

The editor has repeatedly verified this key-note of Lachesis. It is one of the most reliable indications in the materia medica.

The editor once treated a baby for starting when on the point of falling to sleep. Several remedies were given without benefit. He resolved to watch the patient, and, if possible, discover the correct indications for a remedy. It was observed that the child, which was excessively drowsy, would fall to sleep, sleep for about twenty seconds, and then would be seized with a sort of general convulsion of the whole system, which would rouse it from sleep with much crying and weeping. Then it would again fall to sleep, with a repetition of the same occurrences, and so on, during the entire day and night. Lachesis was now given, when the whole series of symptoms subsided in the course of an hour, the child slept peacefully the whole night, and there has never been any return of the trouble.

Restlessness is one of the most characteristic indications for Arsenicum. Still other remedies have it. Here are some other notes taken from the editor's note-book :

Natrum-carbonicum and Phosphorus have restlessness from attacks of anxiety during a thunder-storm (Dr. Carleton Smith).

Carbo-vegetabilis, restlessness and anxiety worse from 4 to 6 P. M.

Eupatorium-perfoliatum, restless with inability to keep still a moment, though he has great desire to do so.

Ammonium-carbonicum, restlessness of the legs.

Sepia has restlessness of the legs with formication in them.

Zinc has intense restlessness of the legs. The editor once cured a case of abscess of the mastoid portion of the temporal bone in a child with Zinc, having been led to the study of this remedy by observing the peculiar restlessness of the legs.

China has restlessness of the legs; must draw them up.

Kali-hydriodicum has restlessness and throwing himself violently about the bed.

Hepar has restlessness of children and unconsciously throwing themselves about the bed. The same symptom occurs under Ignatia.

Jatropha, restlessness. The patient writhes about the bed.

Calcarea-carbonica, restlessness. The patient throws himself about the bed with snoring and groaning all night.

Iodium, restlessness. The patient sits up in bed and throws himself upon it.

Capsicum, restlessness at night. Can't get a comfortable position to lie still one minute.

Ferrum and Pulsatilla, restlessness compels him to walk slowly about. This is Dr. Lippe's key-note for Pulsatilla.

Belladonna, restlessness. Is obliged to move the body to and fro, especially the hands and feet.

Kali-carbonicum, restlessness. Must get up out of bed and walk from sharp stitching pains in loins reaching to the buttocks.

Platinum, general restlessness and fidgety limbs, worse from any attempt to rest. Restlessness from colic, with turning in every possible direction in vain effort to find relief.

See Dr. Knerr's *Repertory to the Guiding Symptoms* for more of this kind of indications.

